# Prevalence and associated factors for hepatitis B infection among pregnant women attending antenatal clinic at SOS Hospital in Mogadishu, Somalia

**DOI:** 10.3389/fgwh.2024.1279088

**Published:** 2024-05-21

**Authors:** Shafie Abdulkadir Hassan, Yousif Mousa Alobaid Ahmed, Babiker Saad Almugadam, Yahye Sheikh Abdulle Hassan

**Affiliations:** ^1^ Department of Microbiology, Faculty of Medical Laboratory Sciences, University of El Imam El Mahdi, Kosti, Sudan; ^2^ Department of Medical Laboratory Sciences, Faculty of Medicine and Health Sciences, Jamhuriya University of Science and Technology, Mogadishu, Somalia; ^3^ Department of Nursing and Midwifery, Faculty of Medicine and Health Sciences, Jamhuriya University of Science and Technology, Mogadishu, Somalia

**Keywords:** hepatitis B virus, hepatitis B surface antigen, pregnancy, associated factors, Mogadishu city

## Abstract

**Background:**

Hepatitis B virus (HBV) remains a leading cause of chronic hepatitis, maternal complications, and neonatal deaths in sub-Saharan Africa. Mother-to-child transmission is a major route of HBV transmission in endemic areas. This study aimed to determine the prevalence of hepatitis B infection and its associated factors among pregnant women attending Antenatal Care clinics at SOS Hospital in Mogadishu, Somalia.

**Methods:**

The research followed a cross-sectional design, and the participants were chosen through systematic random sampling, including every fifth outpatient. Each participant provided a blood sample for standard testing, and their consent was obtained before conducting Hepatitis B screening using the ELISA method.

**Results:**

In our study of 384 pregnant women, 43 individuals (11.2%) tested positive for HBsAg. The frequency of HBsAg seropositivity was significantly higher in subjects with no education when compared to those with primary education (AOR = 0.1, 95% CI: 0.01–0.96, *p* = 0.046). Caesarian Section (AOR = 0.02, 95% CI: 0.004–0.0103, *p* = 0.001), blood transfusion (AOR = 11.6, 95% CI: 3.44–38.08, *p* = 0.001), previous dental procedures (AOR = 0.1, 95% CI: 0.04–0.38, *p* = 0.001), and unsafe injections in the past (AOR = 0.3, 95% CI: 0.09–0.91, *p* = 0.035) were identified as significant risk factors for hepatitis positivity.

**Conclusions:**

The study found a higher prevalence of hepatitis B compared to previous studies. Factors such as blood transfusion, dental procedures, Caesarian Section, and unsafe injections were associated with hepatitis B infection. It is essential to raise awareness, promote preventive measures, and implement routine screening for pregnant women so as to stop the transmission of hepatitis B to their children.

## Background

Hepatitis B Virus is an emerging global health problem (1). Approximately 65 million women of reproductive age groups worldwide are infected with hepatitis B virus and 9 in every 10 mothers with hepatitis B infection transmit the diseases to their child's at the time of birth (2). Hepatitis B is widespread in Africa, affecting 7%–26% of people (3). The regions with the highest prevalence are mainly in Sub-Saharan Africa (4). As a part of Sub-Saharan region, Somalia is classified among the countries as having a high hepatitis B surface antigen (HBsAg) endemicity of more than 8% (5, 6). This indicates HBV infection among pregnant women becomes public health problem in the country. Previous studies have found different factors associated with of HBV infection in pregnant women, these include having multiple sexual partners, dental procedure history, admission history to health facilities, genital mutilation, abortion history, history of giving birth by traditional birth attendants, and blood transfusion (7, 8). The Hepatitis B virus (HBV) can be transmitted through vertical transmission from mother to child (9), and various interventions have shown efficacy in reducing this transmission. These interventions include the use of antiviral drugs during the last trimester if the viral load exceeds 10 million copies per milliliter, as well as administering both immunoglobulin and antigen to the newborn within 24 h of birth, which has demonstrated positive outcomes (10). Despite the absence of published data over the past two decades, this research provides essential insights into the prevalence and risk factors of Hepatitis B among pregnant women in Somalia, aiming to inform targeted public health strategies and ultimately reduce the burden of the disease in this vulnerable population. Therefore, this study aimed to determine the prevalence of HBV infection and associated risk factors among pregnant women attending SOS Hospital in Mogadishu, Somalia.

## Methods

This is a cross-sectional study conducted at SOS Hospital in Mogadishu, Somalia from March 25, 2022, to June 5, 2022. The study focused on pregnant women receiving antenatal care (ANC) at the hospital.

### Inclusion and exclusion criteria

The inclusion criteria for this study included any pregnant woman attending SOS hospital during the study period who had not been vaccinated against hepatitis B and did not know her hepatitis B status. Conversely, the exclusion criteria encompassed any pregnant woman attending SOS hospital who had been vaccinated against the hepatitis B virus or had previously tested positive for any hepatitis B seromarkers.

### Sample size determination

The sample size (*N*) was determined through manual calculation. The estimated proportion of exposure was 0.5 (*p*) for both exposed and unexposed groups. A 95% confidence interval was used with a confidence estimate of 1.96 (*Z*) and a precision of 0.05 (*d*). A design effect of 1 was applied. The sample size was obtained using the formula mentioned below (11)

$$N=Z^{2}pq/d^{2}$$

$$N=\frac{{\left(1.96\right)}^{2}\;*\;0.5\;*\;05}{0.05^{2}}$$

$$N=\frac{3.8416\;*\;0.25}{0.0025}$$

N=384

Tools and techniques used to collect the Data.

The data were collected using a structured questionnaire that included details about sociodemographic characteristics, health-related factors, practices, and behavioral variables related to health. Data collectors and supervisors underwent training before data collection, and the collected data were checked daily for completeness by the supervisor and principal investigator.

### The detection of HBsAg using the enzyme-linked immunosorbent assay (ELISA) followed by Sandwich principle

5 ml of blood was collected from each participant, and the serum was separated from the blood by centrifugation at 3,000 rpm for 5 min. The manufacturer's instructions were followed to examine the serum samples for the presence of hepatitis B surface antigen using an ELISA kit (LumiQuick, USA) using MR 96A ELISA reader (Mindray, China).

### Data analysis

The data were analyzed using SPSS statistical software (Version 20). For descriptive statistics percentages and frequency were used. Association between prevalence and related categorical variables such as age, gender, education, occupation was analyzed using Chi-square test, with *P* value < 0.05 was considered significant. Binary logistic regression model was used to determine the factors significantly associated with the prevalence of HBV; the results were considered statistically significant at 95% confidence interval (*p* < 0.05). Crude and adjusted odds ratios with 95% CI were used to see the strength of association. Predictor variables with a *p*-value <0.05 in the bivariate analysis were candidates for the multivariable logistic regression model. A *p*-value of less than .05 in multivariable logistic regression was considered statistically significance.

## Results

### Demographic profile of study participants

A total of 384 pregnant woman participated in this study with a response rate of 100%. Respondents age ranged between 16 to 45 years. The majority of respondents, comprising 378 (82.9%), were married, while 6 (1.3%) were divorced. In terms of educational level, 210 (46.1%) had no formal education, 84 (18.4%) had primary education, 65 (14.3%) had secondary education, and 25 (5.5%) of pregnant woman had tertiary education, as shown in Table 1.

**Table 1:** Characteristics and HBV status of a pregnant woman.

Variable	HBV status of mothers				
	Frequency	Positive	Negative	Rate (%) of HBV infection	*P*-value
Age					
16–25	166	22	144	13.3	0.128
26–35	198	17	181	8.6	
36–45	20	4	16	20.0	
Education level					
No education	210	31	179	14.8	0.001
Primary	84	1	83	1.2	
Secondary	65	7	58	10.8	
Tertiary	25	4	21	16.0	
Marital status					
Married	378	42	336	11.1	0.512
Divorced	6	1	5	16.7	
Gravidity					
Primigravida	128	13	115	10.8	0.914
Secondgravida	87	10	77	11.5	
Multigravida	169	20	149	11.8	
Occupation					
Housewife	241	26	215	10.8	0.769
Business	142	17	125	12.0	
Skincare specialist	1	0	1	0.0	
ABO blood group					
A	103	7	96	6.8	0.227
B	37	3	34	8.1	
AB	6	1	5	16.7	
O	238	32	206	13.4	
Overall prevalence				11.2	

### Seroprevalence of HBV among pregnant women

Out of the 384 pregnant women included in the study, 43 tested positive for HBsAg, resulting in an overall prevalence of 11.2%. The age group between 36–45 had the highest prevalence of Hepatitis B virus (20.0%), while mothers with no education showed the highest occurrence rate of HBV infection (14.8%), compared to those with primary education as shown in Table 1.

### Risk factors of hepatitis B virus infection

The findings suggest that primary education may be associated with a decreased odds of HBV infection compared to no education (AOR = 0.1, 95% CI: 0.01–0.96, *p*-value = 0.046). Women with history of blood transfusion was found to have 11.6 times more likely to test positive for HBsAg compared to those without a history of blood transfusion (AOR = 11.6 (95% CI: 3.44–38.08 with *p*-value = 0.001). Pregnant women who did not have a caesarian section were 98% less likely to be positive for HBsAg than those who did have a caesarian section (AOR = 0.02 (95% CI: 0.004–0.103 with *p*-value = 0.001). Pregnant women with no history of dental procedures were 88% less likely to test positive for HBsAg compared to those with a history of dental procedures (AOR =  0.1 (95% CI: 0.04–0.38 with *p*-value = 0.001). On the other hand, the study found that women who had a history of unsafe injection were 80% less likely to test positive for HBsAg compared to those who had no history of unsafe injection (AOR = 0.3, 95% CI: 0.09–0.91, *p*-value = 0.035), as shown in Table 2.

**Table 2:** Hepatitis B and associated factors among pregnant woman.

Exposure variable	Category	HBsAg positive	HBsAg negative	COR (95% CI)	*P*-Value	AOR (95% CI)	*P*-Value
Educational Level	No Education	31	179	1		1	
	Primary	1	83	0.1 (0.01–0.52)	0.009	0.1 (0.01–0.96)	0.046
	Secondary	7	58	0.7 (0.29–1.67)	0.417	0.9 (0.18–4.210)	0.855
	Tertiary	4	21	1.1 (0.35–3.42)	0.869	0.5 (0.09–2.81)	0.430
History of Blood transfusion	Yes	21	38	7.6 (3.8–15.12)	0.001	11.6 (3.44–38.08)	0.001
	No	22	303	1		1	
History of Caesarian Section	Yes	40	63	1		1	
	No	3	278	0.02 (0.01–0.06)	0.001	0.02 (0.004–0.103)	0.001
History of dental procedure	Yes	37	41	1		1	
	No	6	300	0.02 (0.01–0.06)	0.001	0.1 (0.04–0.38)	0.001
History of FGM	Yes	40	317	1			-
	No	3	24	0.9 (0.29–3.44)	0.99	-	
History of unsafe injection	Yes	20	91	1		1	0.035
	No	23	250	0.42 (0.2–0.79)	0.008	0.3 (0.09–0.91)	

## Discussion

In this study, we assessed the prevalence of hepatitis B virus and associated factors among pregnant women receiving antenatal care in SOS hospital in Mogadishu, Somalia. The prevalence of HBsAg in pregnant women in the current study was 11.2%, surpassing the WHO cut-off level of endemicity at 8%, indicating a highly endemic region. Lack of the history of blood transfusion, dental procedures, Caesarian Section, and unsafe injections have found statistically significant associations with lower hepatitis B virus seropositivity. This finding is higher than with previous studies from Somalia and Ethiopia, that revealed the prevalence of hepatitis B virus of 5.7% and 9.2% respectively (5, 12). But it was lower than studies conducted in Uganda and Ethiopia with prevalence of 11.8%, 11.3% respectively. The difference between studies may result from variations in the study's design, sample size, study populations' geographic locations, and sociocultural variables. On the other hand, similar studies in similar sample populations, across the world found greater seroprevalence of HBV. Our study revealed that the levels of education were found to be associated with HBV infection among mothers, which is consistent with previous studies (2, 13–14). History of blood transfusion emerged as a significant risk factor for HBV infection among pregnant women, this is consistent with findings from Mogadishu, Somalia (15). This could be attributed to the lack of standardization in blood collection and storage practices, which potentially lead to the use of infected blood in transfusions. Similarly, mothers who had undergone cesarean sections or dental procedures were more likely to test positive for HBsAg, aligning with previous research conducted in Ethiopia (7). Lastly, a history of unsafe injection was significantly associated with HBV infection among pregnant women, which is consistent with studies conducted in Ethiopia and Gambia (9, 10).

### Limitations

The study was conducted in a single health facility, which means that the findings may not accurately represent all pregnant women in the Mogadishu area. HBV markers such as HBeAg and HBV-DNA were not detected in this study due to a lack of laboratory setup.

## Conclusion

The prevalence of hepatitis B in this study is higher compared to some previous studies. The factors associated with hepatitis B infection include blood transfusion, history of dental procedures, Caesarian Section, and history of unsafe injection. To reduce the high prevalence found in this study, it's important to regularly test for HBV during pregnancy checkups. Using clean needles and ensuring sterilized equipment are also crucial. These steps can help lower the risk of spreading hepatitis B. It's also essential to raise awareness about these measures to protect against hepatitis B effectively.

## Data Availability

The original contributions presented in the study are included in the article/Supplementary Material, further inquiries can be directed to the corresponding author.
